# Quality of life and healthcare service utilization among methadone maintenance patients in a mountainous area of Northern Vietnam

**DOI:** 10.1186/s12955-017-0633-9

**Published:** 2017-04-20

**Authors:** Long Hoang Nguyen, Lan Huong Thi Nguyen, Victoria L. Boggiano, Canh Dinh Hoang, Hung Van Nguyen, Huong Thi Le, Hai Quan Le, Tho Dinh Tran, Bach Xuan Tran, Carl A. Latkin, Nabil Zary, Minh Thuc Thi Vu

**Affiliations:** 1 School of Medicine and Pharmacy, Vietnam National University, Hanoi, Vietnam; 2 Institute for Global Health Innovations, Duy Tan University, Da Nang, Vietnam; 3 University of California, Berkeley School of Public Health, Berkeley, California USA; 4 Authority of HIV/AIDS Control, Ministry of Health, Hanoi, Vietnam; 5 Institute for Preventive Medicine and Public Health, Hanoi Medical University, Hanoi, Vietnam; 6 Provincial AIDS Center, Tuyen Quang, Vietnam; 7 Department of Hepatobiliary Surgery, Vietnam-Germany Hospital, Hanoi, Vietnam; 8 Johns Hopkins Bloomberg School of Public Health, Baltimore, Maryland, USA; 9 Lee Kong Chian School of Medicine, Nanyang Technological University, Singapore, Singapore; 10 Department of LIME, Karolinska Institutet, Stockholm, Sweden; 11 Department of Immunology and Allergy, National Otolaryngology Hospital, Hanoi, Vietnam

**Keywords:** Quality of life, Health service, Utilization, Methadone, MMT, Vietnam, Mountainous

## Abstract

**Background:**

The expansion of methadone maintenance treatment in mountainous areas in still limited and little is known about its health impacts on drug users. This study aimed to examine health-related quality of life (HRQOL) and health care access among patients engaging in methadone maintenance treatment (MMT) in Tuyen Quang, a mountainous province in Vietnam.

**Methods:**

We conducted a cross-sectional survey with 241 patients conveniently recruited in two MMT clinics (Son Duong and Tuyen Quang). EuroQol-5 Dimensions – 5 levels (EQ-5D-5 L) and Visual analogue scale (VAS) were employed to measure HRQOL. Multivariate logistic and tobit regressions were used to determine the factors associated with HRQOL and health care utilization.

**Results:**

The overall mean score of the EQ-5D index and EQ-VAS were 0.88 (SD = 0.20) and 81.8% (SD = 15.27%), respectively. Only 8.7% utilized inpatient services, and 14.9% used outpatient services. Being more highly educated, suffering acute diseases, and using health service within the last 12 months were associated with a decreased EQ-5D index. Individuals who were multiple substance abusers and those who recently had inpatient care were more likely to have a lower VAS. Older respondents, those taking their medications at the more impoverished clinic, substance abusers, and individuals who were struggling with anxiety/depression or their usual daily activities were more likely to use both inpatient and outpatient care.

**Conclusions:**

In summary, we observed good HRQOL, but high prevalence of anxiety/depression and low rates of service utilization among MMT patients in Tuyen Quang province. To improve the outcomes of MMT services in mountainous areas, it is necessary to introduce personalized and integrative services models with counseling and interventions on multiple substance use.

## Background

People who inject drugs (PWID) has been primary drivers of the HIV/AIDS epidemic in Vietnam, accounting for 60% HIV cases recently [1, 2]. In addition to sharing contaminated needles/syringes during injecting drug, PWID are more likely to engage in HIV-related risk behaviors such as having unprotected sexual intercourse or involving in sex work to gain money for buying illicit drugs [3–5]. These behaviors could lead to the rapid spread of HIV/AIDS epidemic in not only the PWID population, but also the general population [5]. To address this issue, methadone maintenance treatment (MMT) has been commonly used due to its high effectiveness in reducing the frequency of drug use or unsafety sex as well as promoting health service use and enhancing quality of life among drug users [6, 7]. Therefore, the implementation and expansion of MM programs have been considered as a major priority in Vietnam [1].

In the coming years, the Vietnamese Government has strongly committed to enrolling 80,000 illicit drug users in the MMT program [1]. To date, approximately 46.443 patients have enrolled in 251 MMT clinics nationwide, according to the Vietnam Authority for HIV/AIDS Control [8]. However, a rapid cut of international aid to Vietnam in future years will generate obstacles for scaling up the program [9]. This circumstance requires data to optimize resource allocation for further scaling-up of MMT program.

Features such as relapse, dose, treatment adherence, and urine testing are normally used to monitor treatment progress [10–12]. Nevertheless, given the nature of these indicators, they are unable to capture the effectiveness of MMT in terms of health-related quality of life (HRQOL), which are vital to measuring the impact and cost-effectiveness of an intervention or a health care service [13–15]. In the literature, generic instruments such as the WHOQOL-BREF were frequently employed to measure HRQOL [12, 16]. However, their composite scores are restricted and thus might not be as useful in economic evaluations [15]. Meanwhile, the availability of data on health utility and health care utilization during the course of MMT, which is an essential component of a cost-effectiveness analysis, remains limited [13, 15].

In Vietnam, several studies have investigated HRQOL and health care access among MMT patients in urban and rural settings [15, 17]. A study by Tran et al. (2013) in the three largest cities in Vietnam indicated that 63.6% MMT patients utilized health service in the last three months [15]. This utilization rate was much higher than that in general population (with about 40% in the last 12 months) [18]. Additionally, MMT can lead to dramatic improvement in HRQOL and reduction of healthcare utilization and expenditures [15]. Other studies have shown that rural patients are more likely to have a lower HRQOL than their urban counterparts [19]; rural patients also have a higher risk of experiencing catastrophic expenditure (health care expenditure exceeds 10% of total household expenditure) [17]. Nonetheless, there is a scarcity of research among MMT patients in mountainous areas of the country. The objective of this study was to measure HRQOL and health service use and identify their associated factors among MMT patients in Tuyen Quang, a mountainous province in Vietnam.

## Methods

### Study design

A cross-sectional study was carried out in Tuyen Quang province, a mountainous province in Vietnam, from May to August 2016. This province contains six districts. It has one major city with a population of approximately 750,000; 22 ethnic groups are present within the city. There are more than 1,100 illicit drug users in the province, of whom 388 people have enrolled in the MMT program. Currently, there are three MMT clinics. Two of the clinics are in Tuyen Quang City and Son Duong district. The former is representative of the urban setting, and the latter represents a more remote area. The third MMT clinic in Tuyen Quang province is located in Yen Son district, which has only nine patients. Therefore, in this study, we purposively sampled patients from the two primary MMT clinics (Tuyen Quang and Son Duong).

### Study participants

Patients were recruited based on the following criteria: 1) currently taking methadone medication in study sites; 2) available during the study period; 3) 18 years old or above; 4) willing to enroll, and 5) has the ability to answer the questionnaire. First, we invited eligible patients in each of the two clinic study sites to a private room in order to protect their privacy and create a comfortable atmosphere for the interview. Then we explained the purpose of study, as well as the benefits and drawbacks of participation, and gave them the written consent form if they agreed to participate. There were only five female patients available in both clinics, so to ensure confidentiality, we chose instead to recruit 241 male patients for the study.

### Measurements and Instruments

Face-to-face interviews were performed by both master students in public health and substance abuse experts. We did not involve clinic staff from either of the MMT clinics, in order to avoid potential response bias.

The socioeconomic variables of interest were age, educational attainment, marital status, occupation, and ethnicity. Information about household monthly income and spending were also included. The household monthly income was calculated by taking a sum of the annual income of all members of each participant’s household and dividing it by twelve. Then we divided the sample into five categories, from “poorest” to “richest” [17, 20].

To measure HRQOL, we employed an instrument named EuroQol - five dimensions - five levels (EQ-5D-5 L), which includes five elements of HRQOL: Mobility, Self-care, Usual activities, Pain/Discomfort and Anxiety/Depression. Each domain has five levels of responses, from no problems to extreme problems. Combining the responses of the five domains generates a total of 3,125 health states. Using the interim scoring from Thailand’s value set, each state was transformed into a corresponding single index (EQ-5D index) [21, 22]. We used a visual analogue scale (EQ-VAS), with the scores ranging from 0, “The worst health state that you can imagine,” to 100, “The best health state that you can imagine.” The Vietnamese version of the instrument has been validated in prior studies [22, 23]. We also collected patients’ self-reported health status, including their body mass index, HIV serostatus, and whether they were suffering from acute and/or chronic diseases.

Health care service utilization included any in-patient and/or out-patient care that respondents had received during the twelve months prior to the interview (daily outpatient visits for MMT medication were excluded). Patients were asked whether they receive antiretroviral treatment (ART) in conjunction with methadone treatment; patients were also asked about their duration of MMT use.

Substance abuse behaviors were also investigated as potential covariates. We asked patients about drinking practices (measured by the Alcohol Use Disorders Identification Test-Consumption – AUDIT-C), current cigarette smoking, current opioid use, and history of drug rehabilitation.

### Statistical analysis

A *p-*value of <0.05 was used to represent statistical significance. *T*-test, Chi-square and Fisher’s exact tests were used to compare HRQOL and health care access between the two clinics. The missing data were ignored when analyzing the data. The proportion of missing data in the ethnic variable was the highest with 4% (equal to 10 people not reporting). Schafer et al. (1999) and Bannett (2001) indicated that the missing rate of less than 5% did not affect the final result [24, 25]. Therefore, the missing rates in our study are acceptable.

Multivariate logistic and tobit regressions were utilized to identify associated factors. These models were accompanied by a stepwise backward selection strategy and fractional polynomials for the duration of MMT use. The potential markers included in the models were socio-economic characteristics, health status, health care utilization, and risk behaviors. The threshold of *p <* 0.2 for the log-likelihood ratio test was used to produce reduced models.

## Results

Among 241 patients, 51.8% were 40 years of age or older, 53.0% had attained high school education or above, 62.3% were living with their spouse/partner, 47.5% were self-employed and 92.2% were belonged to Kinh ethnic. Regarding risk behaviors, 18.3% patients were hazardous drinkers, 75.7% were current smokers, and 13.4% were current drug users (Table 1).

**Table 1:** Demographic and risk behaviors of respondents

Characteristics	N	%
Age group		
< 30	13	5.4
30 – 40	103	42.7
41 – 50	90	37.3
> 50	35	14.5
Education		
< High school	111	47.0
High school	106	44.9
> High school	19	8.1
Marital status		
Single	54	22.9
Living with spouse/partner	147	62.3
Divorced/widow	35	14.8
Occupation		
Unemployed	15	6.4
Self-employed	112	47.5
Worker/Famer	38	16.1
Other	71	30.1
Ethnicity		
Kinh	213	92.2
Other ethnicity	18	7.8
Risk behaviors		
Hazardous drinker	44	18.3
Current smoker	174	75.7
Current drug user	31	13.4

Table 2 indicates that less than 15% respondents suffered problems in mobility, self-care, usual activities; 19.9% endorsed Pain/Discomfort and 25.9% had Anxiety/Depression. The mean score of the EQ-5D index was 0.88 (SD = 0.20) and EQ-VAS was 81.84 (SD = 15.27). Additionally, 22.0%, 16.2% and 25.5% patients had acute diseases, chronic diseases, and HIV seropositive, respectively.

**Table 2:** Health status and Health-related quality of life among respondents

Characteristics	Tuyen Quang		Son Duong		Total		*p-*value
	N	%	N	%	N	%	
EQ5D5L							
Having problems in mobility	25	15.1	8	11.4	33	14.0	0.46
Having problems in self-care	21	12.7	4	5.7	25	10.6	0.11
Having problems with usual activities	29	17.5	5	7.1	34	14.4	0.04
Pain/Discomfort	39	23.5	8	11.4	47	19.9	0.03
Anxiety/Depression	52	31.3	9	12.9	61	25.9	<0.01
Having acute diseases	36	21.6	17	23.0	53	22.0	0.81
Having chronic diseases	28	16.8	11	14.9	39	16.2	0.71
HIV positive	32	19.9	27	38.6	59	25.5	<0.01
BMI categories							
Underweight	24	14.7	5	7.3	29	12.5	0.25
Normal	130	79.8	61	88.4	191	82.3	
Overweight and obese	9	5.5	3	4.4	12	5.2	
	Mean	SD	Mean	SD	Mean	SD	*p-*value
EQ5D - index	0.87	0.21	0.93	0.17	0.88	0.20	0.04
EQ VAS	82.35	16.50	80.67	12.06	81.84	15.27	0.46

Table 3 reveals that within the last 12 months, less than 10% and 15% of participants utilized inpatient and outpatient services, respectively. Most of patients selected district hospitals for outpatient care (61.1%) and provincial hospitals for inpatient care (71.4%). About 22.8% participants were receiving ART. The mean duration of MMT among sample was 15.3 months (SD = 8.7 months).

**Table 3:** Health service use among respondents within the last 12 months

Characteristics	Tuyen Quang		Son Duong		Total		*p-*value
	N	%	N	%	N	%	
Used inpatient services	20	12.0	1	1.4	21	8.7	0.01
National Hospital	6	30.0	0	0.0	6	28.6	1.00
Province Hospital	15	75.0	0	0.0	15	71.4	0.29
District Hospital	0	0.0	1	100.0	1	4.8	0.04
Community Health Center	0	0.0	0	0.0	0	0.0	-
Private Clinic	1	5.0	0	0.0	1	4.8	1.00
Other	0	0.0	0	0.0	0	0.0	-
Used outpatient services	8	4.8	28	37.8	36	14.9	<0.01
National Hospital	2	25.0	4	14.3	6	16.7	0.60
Province Hospital	0	0.0	5	17.9	5	13.9	0.57
District Hospital	5	62.5	17	60.7	22	61.1	1.00
Community Health Center	0	0.0	2	7.1	2	5.6	1.00
Private Clinic	2	25.0	2	7.1	4	11.1	0.21
Other	0	0.0	1	3.6	1	2.8	1.00
Initial health facility use when becoming ill							
National Hospital	0	0.0	3	5.2	3	1.4	<0.01
Province Hospital	101	66.0	7	12.1	108	51.2	
District Hospital	9	5.9	27	46.6	36	17.1	
Community Health Center	17	11.1	20	34.5	37	17.5	
Private Clinic	25	16.3	0	0.0	25	11.9	
Self-treatment	1	0.7	1	1.7	2	1.0	
Receiving ART	28	17.1	25	36.2	53	22.8	<0.01
Duration of MMT (months), mean, SD	16.4	9.2	12.7	6.7	15.3	8.7	0.03

Reduced multivariate Tobit regression models are shown in Table 4. People who were more well-educated, those with acute disease, and individuals who received inpatient and outpatient care in the last twelve months had a lower EQ-5D index. Patients who were self-employed, those who belonged to the richest quintile group, and those who had a normal BMI had a higher EQ-5D index. Regarding EQ-VAS, patients had a higher VAS if they had higher incomes and if they were receiving ART treatment. Conversely, people who were substance abusers and those who had received inpatient care were more likely to have a lower VAS.

**Table 4:** Factors associated with quality of life among respondents

Characteristics	EQ5D - index		EQ VAS	
	Coef	95% CI	Coef	95% CI
Age group (vs < 30 years)				
> = 30 & < 40 years	0.12	–0.02; 0.26		
Education (vs < High school)				
High school	–0.16*	–0.30;–0.01	3.59	–1.00; 8.19
Above high school	–0.39*	–0.66; –0.12		
Occupation (vs Unemployed)				
Self-employed	0.18*	0.05; 0.32		
Income quintiles (vs Poorest)				
Rich	0.16	–0.02; 0.33	6.40*	0.21; 12.58
Richest	0.26*	0.06; 0.46	12.05*	5.58; 18.52
Location (Son Duong vs Tuyen Quang)	0.12	–0.06; 0.29	–3.53	–8.72; 1.67
Body mass index (vs Underweight)				
Normal	0.35*	0.20; 0.50		
Having acute disease (Yes vs No)	–0.20*	–0.36; –0.05		
Using multiple substances (vs None)				
Abusing one substance	–0.11	–0.24; 0.03	–9.92*	–16.01; –3.83
Abusing two or more substances			–5.84	–13.07; 1.38
Episode of drug rehabilitation (vs None)				
>2 times	0.12	–0.04; 0.27		
Receiving ART (Yes vs No)			7.52*	2.03; 13.02
Duration of MMT (months)	0.00	–0.01; 0.01	0.01	–0.26; 0.28
Receiving inpatient care (Yes vs No)	–0.53*	–0.75;–0.31	–26.66*	–34.67; –18.65
Receiving outpatient care (Yes vs No)	–0.20*	–0.40;–0.01		

* *p <* 0.05

Table 5 shows the factors associated with inpatient and outpatient service utilization. Older respondents, those who used two or more substances, those who had problems with their usual activities, and individuals suffering from anxiety/depression were more likely to use inpatient services. However, those who were employed and those who had one-time drug rehabilitation were less likely to use inpatient services.

**Table 5:** Factors associated with health care service utilization among respondents

Characteristics	Inpatient service use		Outpatient service use	
	OR	95% CI	OR	95% CI
Age	1.26*	1.12; 1.42		
Education (vs < High school)				
High school	2.60	0.61; 11.00		
Occupation (vs Unemployed)				
Employed	0.10*	0.02; 0.57		
Location (Son Duong vs Tuyen Quang)	0.06	0.00; 1.31	32.74*	9.65; 111.06
Using multiple substances (vs None)				
Abusing one substance			2.56	0.70; 9.31
Abusing two or more substances	6.10*	1.04; 35.75	4.83*	1.05; 22.13
Number of times in rehabilitation (vs None)				
One time	0.17*	0.03; 0.95		
Having problems in self-care (Yes vs No)			0.10	0.01; 1.24
Having problems in usual activities (Yes vs No)	8.75*	1.30; 58.96	9.19*	1.51; 55.93
Anxiety/Depression (Yes vs No)	8.53*	1.23; 59.33		
Receiving ART (Yes vs No)			0.21*	0.08; 0.55
Duration of MMT (months)	1.05	0.98; 1.13	1.08*	1.01; 1.15

* *p <* 0.05

Receiving medicine at Son Duong clinic, abusing two or more substances, having problems with usual daily activities, and being on MMT for a longer duration were factors associated with increased outpatient health care utilization, while receiving ART was associated with decreased use of outpatient care.

## Discussion

Our paper provides new information about HRQOL and health care service use among patients being treated for opioid dependence in developing countries. In this study, we found a high level of HRQOL and a low degree of health service utilization among MMT patients in a mountainous setting in northern Vietnam. In addition, the multi-level predictors we identified significantly help maximize the outcomes for patients on MMT, which is vital for the scaling-up of the MMT program in Vietnam.

EQ-5D-5 L is an indirect-preference instrument for assessing HRQOL and EQ-5D index is a composite index that is constituted by five domains in the instrument [12]. Meanwhile, EQ-VAS is a preference instrument that directly measures the perception of patients about their health status [21]. A meta-analysis of Bach et al. (2015) indicated that in the short-term, the HRQOL might be effected by the hope of patients for treatment, while in the long-term, the patients could evaluate their health status more accurately [26]. These authors also suggested the combination between direct and indirect instruments when measuring HRQOL [27]. In this study, we employed both EQ-5D-5 L and EQ-VAS to measure the HRQOL as well as become the dependent variables in the regression models. This strategy would help us to identify associated factors for both short-term and long-term changes of HRQOL among MMT patients.

Overall HRQOL of the MMT patients in our study was higher than those of MMT patients in previous studies [15]. In addition, we found HRQOL in our study to be higher than that in the general population or in other groups, namely HIV positive patients [23, 28]. However, the regression model shows that there was no association found between EQ-5D index and VAS scores and the duration of methadone treatment. This finding is different from a prior study conducted with MMT patients in urban and rural settings in Vietnam [19]. The previous study explained that patients who are being treated for a long period become concerned not only with their health status, but also with social, interpersonal, and economic issues such as stigmatization, occupations, and income [14, 19, 26, 29]. Small sample size might be the reason that we could not draw similar conclusion. Therefore, a further longitudinal study with larger sample size should be conducted to identify the relationship between HRQOL and duration of MMT.

We found that several socio-economic factors influenced MMT patients’ HRQOL. For example, having a job and a higher income were positively associated with a higher HRQOL, which is similar to previous observations in Vietnam and in other countries [14, 19, 30]. However, we also found the higher education was a considerable marker of reduction in HRQOL. It is different from the previous study in urban and rural settings, which found that higher education had a positive association with higher HRQOL [19]. It is noteworthy that the association between socio-economic characteristics and health status can be affected by other interactions within the physical and social environment [31]. The current study was conducted in Tuyen Quang, a mountainous province in Vietnam, where 22 ethnic minorities were living in a wide geographical area with mostly high mountains, hills and deep valleys. Despite the current availability of three MMT clinics in Tuyen Quang, it might be difficult for patients to access these clinics because of the geographical barriers, especially those having high education with stable jobs. Some studies suggest that well-educated people are more likely to have depression/anxiety than other groups, leading to a decrease in HRQOL [27, 32–36]. Visiting clinics to take medication daily wastes their time and affect their job; hence, they might suffer more psychological distress than other people. Bach et al. measuring the HRQOL of general populations in the mountainous areas suggested that higher distance to the heath station was related to the lower HRQOL [28].

This study also reaffirms the necessity of noticing MMT patients’ nutritional status, physical health, and substance use patterns, which has been highlighted in other studies. Patients with normal BMI had higher HRQOL compared to other groups [37, 38]. Conversely, respondents experiencing acute diseases or receiving inpatient/outpatient services in the last twelve months had lower HRQOL [28]. In addition, the availability of at least one behavior such as smoking, drug use or alcohol use led to declining HRQOL among MMT patients [14, 19].

In this study, we found that only 8.7% and 14.9% of MMT patients use inpatient and outpatient services, respectively. These rates were consistent with previous studies done with MMT patients in Vietnam [17]. A study by Tran et al. in HIV-positive patients taking methadone treatment suggested that MMT might decrease the frequency of utilizing health services, and therefore reduce the risk of suffering a catastrophic health care expenditure [15].

Nonetheless, because our study was conducted in a mountainous area, the low proportion of respondents using health services might result from the long distance between patients’ living places and the health facilities. In the literature, distance has been identified as a great barrier to health care access, especially in mountainous settings [39–41]. We observed that the distance from MMT clinics to the nearest district hospital was about 10–12 km. In addition, poor quality of health care services might also contribute to underuse [28]. A commune health center (CHC) is the nearest health care level that patients can access in their communities [42]. However, only 5.6% of patients used outpatient services in their CHC, while 61.1% used services in district hospitals. In addition, no one used inpatient services in their CHC. An evaluation by Nguyen et al. in remote and poor areas in Vietnam suggests that the utilization rate improved sustainably after enhancing the quality of services [43]. Notably, patients in Son Duong district, a more underserved district, were more likely to use outpatient services, compared with those living in Tuyen Quang city. This result might suggest the importance of decentralization of MMT program in the mountainous setting.

There are several implications that could be drawn from the study. First, the high proportion of current illicit drug users requires further interventions to promote the medication adherence among MMT patients. Additionally, health staffs should counsel substance use disorders for tobacco and alcohol as well as nutrition care to improve patients’ healthy lifestyles and boost their HRQOL [19, 44]. Second, integrating general and metal health care into MMT clinics should be considered that could provide care to patients timely if they suffered illness or depression during treatment. Third, providing vocational training to the patients and introducing job opportunities to raise their income are potential to increase the patients’ HRQOL. Finally, decentralizing MMT programs, as well as strengthening the capacities of grass-roots health care services such as local CHCs, might have the potential to improve health care provision for MMT patients in Tuyen Quang province, which can promote the health care use among MMT patients.

This study is among the first to look at behavior patterns and HRQOL among MMT patients in mountainous parts of Vietnam. Employing the EQ-5D-5 L, a validated and internationally recognized instrument, helps to improve the reliability and reproducibility of our findings. However, some limitations should be noted. First, it is difficult to use a cross-sectional to draw causal conclusions, and therefore we require an additional longitudinal study to clearly define the relationships between MMT and HRQOL, as well as health care use, among MMT patients in mountainous parts of Vietnam. Second, patients’ recall bias may lead to under- or overestimation of some responses and therefore may bias our findings. Third, our convenience sampling procedure with a small sample size restricts the generalizability of study’s results for the population of Vietnam as whole. Lastly, other clinical and social factors such as adherence, methadone dose, and stigma were not included in the study. Further studies should be conducted to address these limitations and improve MMT program planning in Vietnam.

## Conclusion

In summary, we observed a high level of HRQOL and an overall low rate of health care utilization among MMT patients in Tuyen Quang province, a mountainous area in northern Vietnam. Integrating and decentralizing MMT clinics accompanied by strengthening capacities for grass-roots health care services should be considered when implementing MMT in Vietnam, especially in mountainous areas.

## Abbreviation

AIDS: Acquired immune deficiency Syndrome
ART: Antiretroviral therapy
AUDIT-C: Alcohol use disorders identification test – consumption
BMI: Body mass index
EQ-5D-5 L: EuroQol – 5 dimensions – 5 levels
HIV: Human immunodeficiency virus
HRQOL: Health-related quality of life
MMT: Methadone maintenance treatment
PWID: People who inject drugs
VAS: Visual analogue scale
